# Assessment of community-acquired urinary tract infections treatment in the emergency department: a retrospective study

**DOI:** 10.3389/fcimb.2024.1433597

**Published:** 2024-10-16

**Authors:** Marie Ange Ghaleb, Antoine Zoghbi, Zeina Bou Chebl, Eddy Lilly, Gebrayel Saliba, Jacques Choucair, Racha Ibrahim

**Affiliations:** ^1^ Emergency Department, Hôtel Dieu de France Hospital, Beirut, Lebanon; ^2^ Infectious Diseases Department, Hôtel Dieu de France Hospital, Beirut, Lebanon; ^3^ Urology Department, Hôtel Dieu de France Hospital, Beirut, Lebanon

**Keywords:** urinary tract infection, emergency department, extended spectrum beta-lactamase, broad spectrum antibiotic, guidelines, stewardship

## Abstract

**Introduction:**

Urinary tract infection (UTI) is one of the most common medical complaints in the emergency department (ED). The aim of this study was to assess the real indication of an initial broad-spectrum treatment administered in the ED for hospitalized patients with a diagnosis of community-acquired UTI (CAUTI).

**Materials and methods:**

This is a monocentric observational retrospective study conducted in the ED of one of the largest tertiary care centers in Lebanon, on a two-year period, including adult patients admitted to the hospital for a CAUTI. The primary outcome was to evaluate the need of downgrading empirical antibiotherapy started in the ED. Secondary outcomes included a description of CAUTIs characteristics: prevalence and risk factors for (extended spectrum beta lactamases) ESBL-related infection, complicated and uncomplicated UTIs, empirical and targeted treatment, and finally the rate of adherence to local guidelines.

**Results:**

The most isolated strains on urine cultures were gram negative bacilli (GNB) with 29.1% producing ESBL; 69.4% of patients received an ESBL-targeting empirical treatment in the ED, in agreement with local guidelines, 46% of which needed a downgrade. Amikacin adjunction was only indicated in 42.8% of the cases. Patients who received antibiotics in the last 6 months had a 2.36 times higher risk of developing an ESBL-related infection.

**Conclusion:**

This study showed a high adherence rate to local recommendations suggesting the use of empirical ESBL-targeting antibiotherapy even in uncomplicated UTIs. However, the frequent need of de-escalation highlights the importance of establishing an efficient multi-drug resistant (MDR) bacteria surveillance system in the community in order to elaborate a stewardship program with more solid local guidelines.

## Introduction

Urinary tract infection (UTI) is one of the most common medical complaints in the emergency department (ED). It can vary from a non-complicated cystitis to a complicated pyelonephritis or even urosepsis. It can affect both, women and men, adults and children. Concerning the main pathogens incriminated in UTIs, a retrospective study conducted in multiple tertiary care centers in South Lebanon, over a nine-month period showed that Escherichia coli, Klebsiella pneumoniae and Proteus mirabilis were the main organisms involved in UTIs in patients presenting to the laboratories or the urology department (Sokhn et al., 2020). For patients who need admission to the hospital, initial intravenous antibiotherapy is mainly started by ED physicians, theoretically guided by local epidemiology and guidelines, until microbiological results are available, and then adjusted accordingly. Two studies conducted in two different tertiary care centers in Beirut, Lebanon, showed a prevalence of 14.15% of (extended spectrum beta-lactamases) ESBL-producing gram negative bacilli (GNB) among community-acquired UTIs (CAUTIs) in 2016 (Abi Hanna, 2016) with an increase from 2.3% to 16.8% between 2000 and 2009 (Daoud and Afif, 2011). In addition, another retrospective study conducted in one of the largest hospitals in North Lebanon, revealed an increase of ESBL production in E. coli urinary isolates between 2005 and 2012 (Daoud et al., 2015). Therefore, the Lebanese Society of Infectious Diseases and Clinical Microbiology (LSIDCM) have published in 2017, the national guidelines for UTI management according to these data, thus recommending the use of ESBL-targeting empirical antibiotherapy even in cases of uncomplicated pyelonephritis and prostatitis (Husni et al., 2017).

A retrospective study conducted in the ED of three different hospitals in Lebanon showed 40% of adherence to international guidelines in the management of UTIs (Fahs et al., 2017).However, no studies have evaluated the adequacy of initial antibiotherapy when given on the basis of our local guidelines in Lebanon. Thus, our study aimed to assess the real indication of an initial broad-spectrum treatment administered in the ED for hospitalized patients with a diagnosis of CAUTIs, by measuring the rate of subsequent antibiotic modification based on microbiological results, in order to reduce the inappropriate use of these molecules and to optimize our stewardship programs.

## Materials and methods

This is an observational retrospective study conducted in the emergency department of one of the largest tertiary care centers in Beirut, Lebanon, between September 2019, and September 2021.

This study was approved by the Ethical Committee of the hospital and registered under the number CEHDF 1912.

### Patient selection

The study population was extracted from the Software DxCare by looking for the following ICD (International Classification of Diseases): cystitis, pyelonephritis, urinary tract infection, ureteritis, prostatitis and urosepsis.

We included all patients, males or females, aged 18 years and older that presented to the ED with one of these symptoms: dysuria, hematuria, pelvic or lower back pain, frequency of urination, fever or chills, nausea and vomiting and were diagnosed with CAUTI that needed admission to the hospital, according to the ED physician. The exclusion criteria were: hospitalization in the last six months, urological intervention in the last 3 months, immunosuppressive diseases, and treatments (chemotherapy, immunosuppressants, corticosteroids), renal replacement therapy and pregnancy.

### Data collection

We collected the following data using the patients’ medical records: Age, sex, presence of diabetes, antibiotic use during the last six months, urine culture results, hemodynamic status on presentation to the ED, empirical and targeted antibiotherapy after microbiological results.

### Statistical analysis

Statistical analyses were performed using SPSS version 26 (IBM Corp., Armonk, NY, USA). Descriptive statistics were utilized to summarize the demographic and clinical characteristics of the participants, with continuous variables presented as means and standard deviations and categorical variables as frequencies and percentages.

For analyzing categorical data, the Pearson Chi-Square test was employed to assess the relationships between empirical and targeted antibiotherapy, the occurrence of multi-drug resistant (MDR) organisms, and various demographic and clinical factors. Fisher’s Exact Test was applied where the expected frequencies were low (i.e., less than five in any cell of a contingency table), ensuring the validity of the results in small sample cases.

Cramer’s V was computed to quantify the strength of the associations observed. Odds ratios (ORs) and 95% confidence intervals (CIs) were also calculated to estimate the risk associated with previous antibiotic use. Statistical significance was set at a p-value of less than .05.

### Outcomes

The primary outcome was to evaluate the need of downgrading empirical antibiotherapy started in the ED. Secondary outcomes included a description of CAUTIs characteristics: urine culture results, extended spectrum beta-lactamases (ESBL)-producing gram negative bacilli (GNB) prevalence and risk factors, complicated and uncomplicated infections, empirical and targeted treatment, and finally the rate of adherence to local guidelines.

## Results

Among 585 patients who presented to the ED for a urinary tract infection (cystitis, pyelonephritis, ureteritis, prostatitis) between September 2019 and September 2021 and were admitted to the hospital, only 72 (12.3%) had a CAUTI and were included in the study. Of these, only 4.2% (3/72) had cystitis, 59.7% (43/72) had pyelonephritis, 34.7% (25/72) had prostatitis and one patient (1/72) had ureteritis. The mean age was 59 years ±  25.5, 32 men and 40 women (**Table 1**).

**Table 1:** Patients characteristics and types of UTIs.

Characteristics	N(%)
Total patients	72
Gender	
Males	32 (44.4%)
Females	40 (55.5)
Diabetes	13 (18%)
Age in years	
Mean	59 ± 25.5
Types of UTI	
Cystitis	3 (4.1%)
Pyelonephritis	43 (59.7%)
Ureteritis	1 (1.4%)
Prostatitis	25 (34.7%)
Complications	
Obstruction	17 (23.6%)
Urosepsis	7 (9.7%)

### Choice of empirical antibiotherapy

Almost half of the patients (51.4%, 37/72) received empirical monotherapy, whereas 48.6% (35/72) received empirical combined therapy. The antibiotics used as monotherapy were: ceftriaxone (56.8%, 21/37), piperacillin-tazobactam (24.3%, 9/37), carbapenems (16.2%, 6/37), and oral cefixime for one patient.

As for the combined therapy, the most used combination was amikacin and 3^rd^ generation cephalosporin (3GC),(62.9%, 22/35) followed by amikacin and piperacillin-tazobactam (28.6%, 10/35). Two patients received amikacin with carbapenems, and one patient received amikacin and ciprofloxacin. (**Table 2**). Therefore, a total of 69.4% of patients (50/72) received an ESBL-targeting empirical treatment for CAUTI. There was no correlation between receiving amikacin and isolating ESBL-producing bacteria on urine cultures, Cramer’s v 0.02.

**Table 2:** Empirical antibiotherapy.

Antibiotherapy	N (%)
Empirical monotherapy	37(51.4%)
Ceftriaxone	21(29.2%)
Piperacillin-tazobactam	9(12.5%)
Carbapenems	6(8.3%)
Oral cefixim	1(1.4%)
Empirical combined therapy	35(48.6%)
Amikacin + 3rd generation cephalosporin	22(30.6%)
Amikacin + Piperacillin-tazobactam	10(13.9%)
Amikacine + carbapenems	2(2.8%)
Amikacin + ciprofloxacin	1(13.9%)

### Urine culture results

The most isolated strains were GNB including mainly Escherichia Coli (E.coli) (55.5%, 40/72), Klebsiella pneumoniae (8.3%, 6/72), less frequently Citrobacter Koseri (3/72) and Enterobacter sp (1/72). Twenty-one out of 72 (29.1%) had an ESBL-producing GNB. Three of 72 patients had Pseudomonas aeruginosa, and 2 had enterococcus faecalis. Urine cultures were negative in 16 cases (16/72, 22.2%), with only 4 that had taken antibiotics prior to admission.

A significantly higher percentage (52.20%) of patients who had received antibiotics in the last 6 months had an ESBL-related infection, versus 24.50% of patients who did not receive antibiotics in the last six months. This relation was significant with a strong correlation (Pearson Chi-Square p value.031<.05, Cramer’s V of.274). Patients who received antibiotics in the last 6 months had a 2.36 times higher risk of developing an ESBL-related infection, OR 3.364 95%CI (1.182-9.57).

### Targeted antibiotherapy

Eleven out of 72 (15.2%) patients needed an antibiotic upgrade, whereas 27 (37.5%) benefited from a downgrade or an oral switch mainly to fluoroquinolone or Trimethoprim/sulfamethoxazole.

Of note, 21 patients were maintained on third generation cephalosporin (3GC) but were switched from intravenous to oral form. There was no change of empirical antibiotherapy for 13 out of 72 (18.1%).

Among the patients who received an ESBL-targeting empirical treatment (N=50), 46% (23/50) benefited from a downgrade, 30% (15/50) were maintained on IV ESBL-targeting treatment including 4 patients who were switched from 3GC + amikacin to carbapenems, with no possible oral switch (**Table 3**).

**Table 3:** ESBL-targeting empirical treatment modification.

ESBL-targeting empirical antibiotherapy	N=50	Downgrade N=23
Amikacin+ 3^rd^ generation cephalosporin	22	10
Piperacillin-tazobactam ± Amikacin or Carbapenems ± Amikacin	27	12
Amikacin+ Ciprofloxacin	1	1

### Complicated urinary tract infection and MDR bacteria

**Figure 1:**
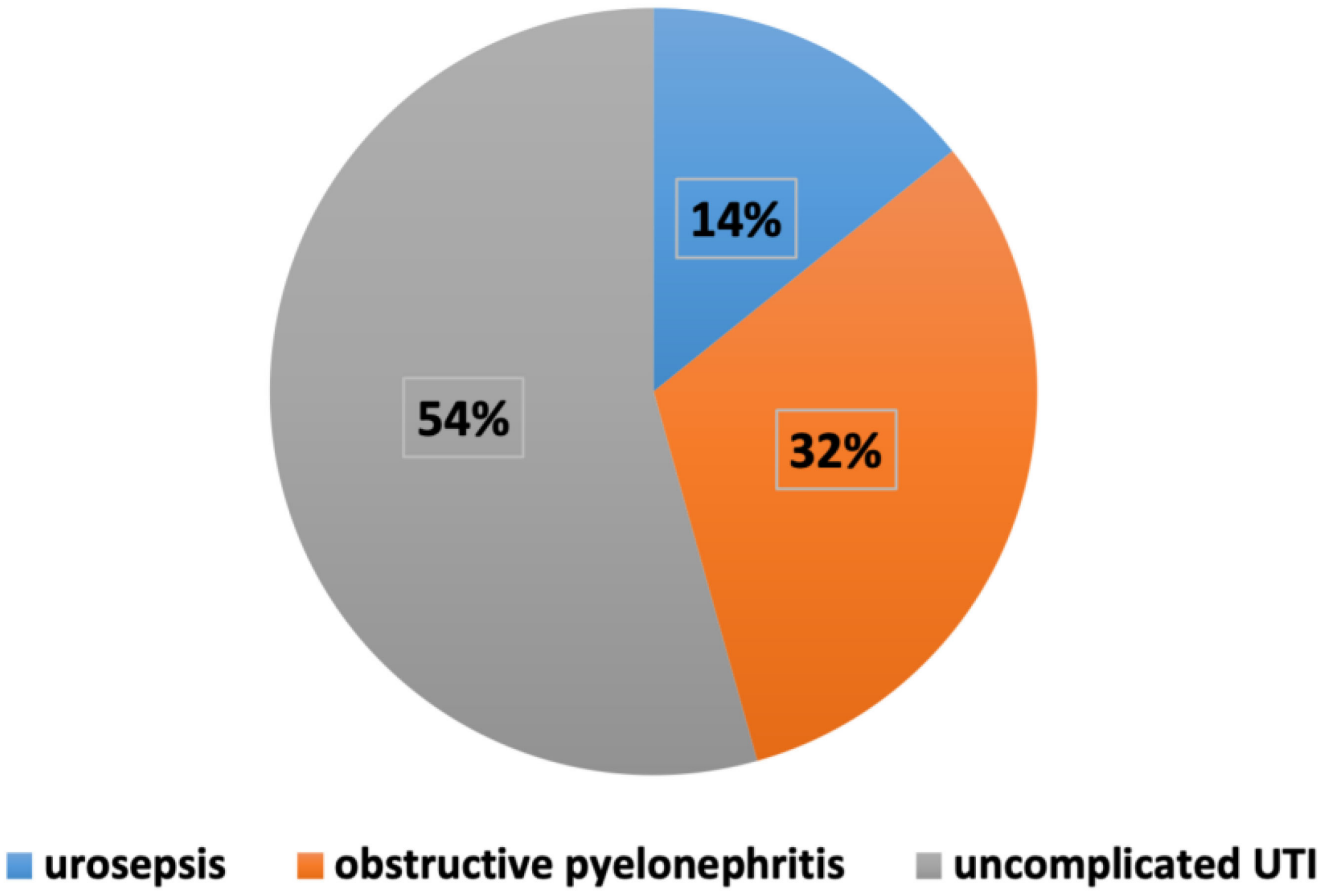
Distribution of complicated and uncomplicated UTIs among patients receiving amikacin-based antibiotherapy.

In this study, complicated UTIs were defined as having one of the following criteria: male sex, urosepsis, diabetes, and/or obstructive pyelonephritis (Uroweb - European Association of Urology, 2024). These patients were two times (59.1%) more prone to receive amikacin-based antibiotherapy in the ED than patients with uncomplicated UTIs (32.1%). This difference was statistically significant and strongly correlated (p.032<.05, Cramer’s V 0.263). However, 5 patients of 35 (14.2%) who received amikacin-based combined therapy had urosepsis, and 11 of 35 (31.4%) had obstructive pyelonephritis (**Figure 1**). An ESBL producing GNB was isolated in 50% of those cases.

**Figure 2:**
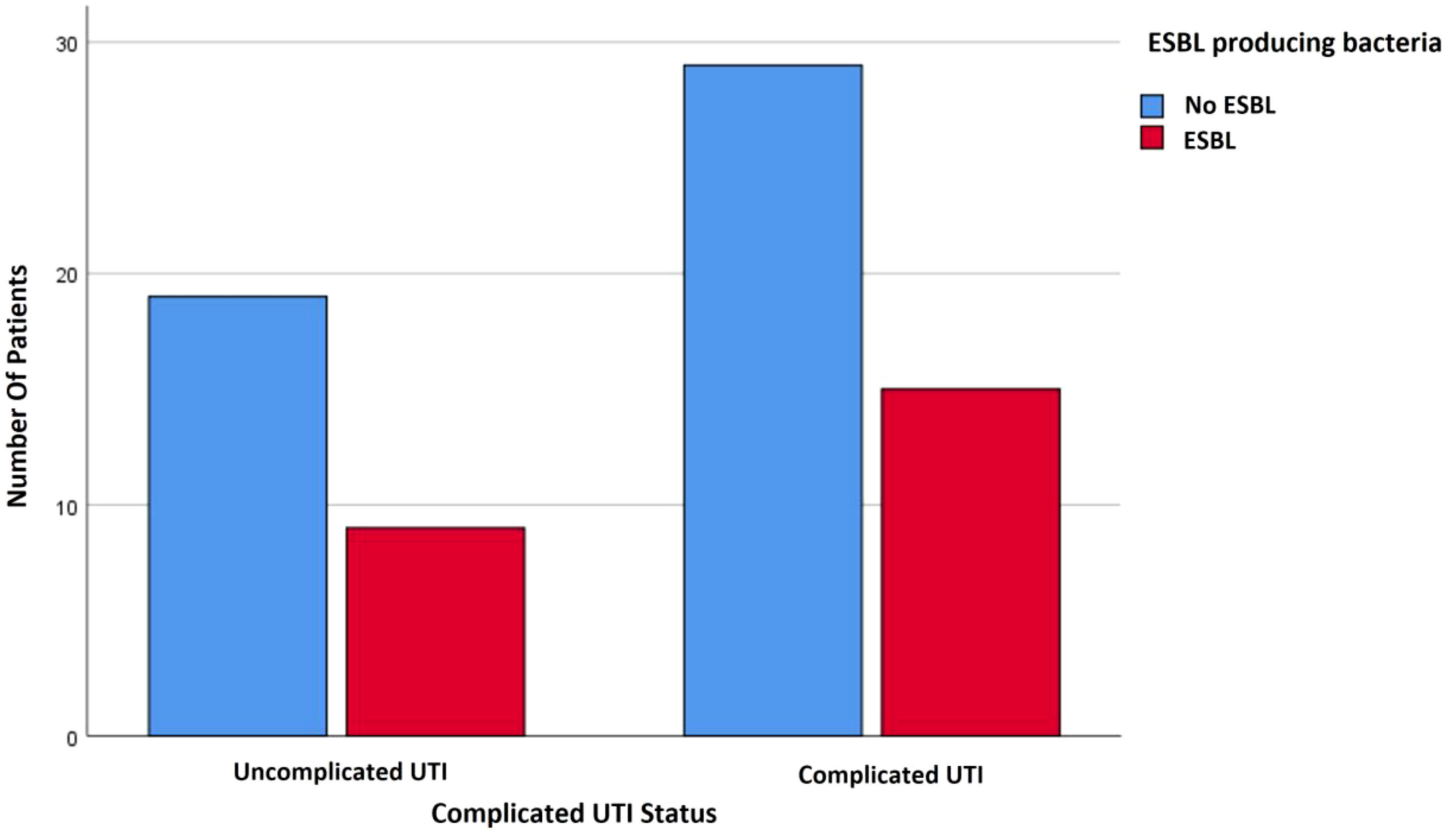
Correlation between complicated UTIs and ESBL-producing bacteria.

There was no significant correlation between isolating an ESBL-producing bacteria and complicated UTIs (**Figure 2**). Thus, 92.31% of the patients who presented with obstructive pyelonephritis had a sensitive strain, whereas only 7.69% of patients with obstructive pyelonephritis had an ESBL producing bacteria. This percentage is statistically significant with a strong correlation (p= 0.048, Cramer’s V of.255).

## Discussion

The aim of the study was to evaluate the need of downgrading empirical antibiotherapy in order to assess the management of CAUTI in the ED of our hospital based on actual available epidemiological data and local guidelines. The types of CAUTIs were mostly pyelonephritis, followed by prostatitis.

We found a tendency of ED physicians to prescribe an ESBL-targeting empirical treatment in 69.4% (50/72), including piperacillin-tazobactam, carbapenems and 3GC with amikacin, even without previously documented ESBL colonization. In fact, a high rate of ESBL-producing GNB (29.1%) was isolated in our urine cultures which is in agreement with the previous studies conducted in Lebanon (Daoud et al., 2015; Abi Hanna, 2016; Sokhn et al., 2020).

Consequently, a combined amikacin-based antibiotherapy was prescribed in 48.6% of these patients on the basis of the 2018 UTIs guidelines of the LSIDCM, though amikacin adjunction was only indicated in 42.8% of the cases (4 patients out of 35 had urosepsis, and 11 out of 35 patients had an obstructive pyelonephritis) according to the IDSA and European guidelines (Infections urinaires recos 2017 - Actualités - Documents - spilf -; Uroweb - European Association of Urology, 2024; Gupta et al., 2011).

Despite the high rate of adherence to the LSIDCM guidelines, a de-escalation was still needed in almost half of the cases (23/50) who received broad-spectrum antibiotherapy at presentation. In addition, even in cases of complicated UTIs justifying the use of broad-spectrum antibiotics or the prescription of aminoglycosides, these cases appeared to be mostly caused by sensitive strains.

The main risk factor for having an ESBL-producing GNB related infection in this study was antibiotic use in the last 6 months. This was also the case in a retrospective study conducted in a tertiary care center in Beirut where antibiotic use in the last 90 days was a predictor of an ESBL UTI infection (Bou Chebl et al., 2021). Knowing that there are other risk factors for having ESBL-producing GNB, we decided to exclude patients with prior hospitalization in the last 6 months, urological intervention in the last 3 months and known urinary colonization with ESBL-producing bacteria in order to better assess the empirical management of CAUTIs in the ED.

In addition, microbiological documentation is crucial for the subsequent narrowing of initial antibiotherapy and switch to oral form, as recommended by all guidelines (Uroweb - European Association of Urology, 2024; Gupta et al., 2011; Husni et al., 2017). However, in this study we noticed that 22.2% of urine cultures were negative, only 4 of which were masked by a prior antibiotic exposure. For the 12 remaining cases, 5 had urosepsis or obstructive pyelonephritis explaining the possible administration of antibiotic treatment before collecting the urinary specimen, but no reasonable explanation was found for the rest. In fact, we noticed that antibiotics might have been started in the ED before collecting cultures by some health care workers, hence, there is a need to continuously spread awareness about the importance of microbiological documentation among practitioners, through an efficient antimicrobial stewardship program (M S. [Antibiotic stewardship], 2023).

In fact, a prospective study conducted in a tertiary care center in Lebanon proved that implementing antimicrobial stewardship programs can help reduce antibiotic use in an acute care setting with a high acceptance rate (Shallal et al., 2022). This program can also be implemented in the ED by collecting detailed regional resistance data in UTIs, communicating with the infectious diseases specialists and pharmacists, designating a reporting structure for continuous enlightenment and education healthcare workers on the importance of stewardship and its application (CDC, 2024).

Our study has some limitations. In fact, it is an observational monocentric study with a small sample size. However, it reflects well the medical practices in the management of CAUTIs in our ED, highlighting the need to reinforce the stewardship program starting with the elaboration of an epidemiologic surveillance network in order to continuously review and update local guidelines. Meanwhile, the use of point-of-care enzymatic tests could be helpful to avoid the overuse of carbapenems or aminoglycosides in the absence of sepsis or recent documented ESBL colonization (Mizrahi et al., 2021).

Another limitation is that our study focuses on ESBL prevalence without assessing the resistance rate to other antibiotic classes including fluoroquinolones and trimethoprim-sulfamethoxazole, first-line oral therapies in UTI management.

More studies are needed to assess the real utility to combine aminoglycosides to 3GC for the treatment of uncomplicated pyelonephritis as recommended in our guidelines, in contrast with the international guidelines, where amikacin is spared for cases of complicated or septic infections.

In addition, the LSIDCM guidelines recommend the use of cefixime as an alternative of oral switch therapies for acute bacterial prostatitis, as shown in our study, despite the absence of this option in other guidelines. In fact, there is no sufficient clinical studies demonstrating the efficacy of oral cephalosporins in acute prostatitis management knowing their low penetration capacity in prostate tissue (Litvak et al., 1976).

However, more studies are needed to evaluate the cure and relapse rates of the UTI after de-escalation of antibiotherapy, which was not done in this study that focused mainly on the choice of adequate empirical treatment.

## Conclusion

Despite its small sample size, this real-world study showed a high percentage of broad-spectrum antibiotic overuse in the ED for the management of community-acquired urinary tract infections, based on local recommendations that suggest targeting ESBL-producing GNB empirically for uncomplicated pyelonephritis and prostatitis. However, the frequent need of de-escalation highlights the importance of establishing an efficient MDR bacteria surveillance system in the community in order to empower our stewardship program through more solid local guidelines.

## Data Availability

Due to confidentiality reasons, the raw data generated and analyzed during this study are not publicly available. The patients involved in this research were treated at a private hospital and are not part of the public healthcare system, making it unsuitable to include their data in a national repository.However, to ensure transparency and facilitate further research, we are willing to provide anonymized data upon request. Access to the data will be granted under specific conditions, ensuring that the privacy and confidentiality of the patients are maintained. Researchers interested in accessing the anonymized dataset are encouraged to contact the corresponding author, outlining their request and the intended use of the data.
